# Viewing the global health system as a complex adaptive system – implications for research and practice

**DOI:** 10.12688/f1000research.126201.1

**Published:** 2022-10-07

**Authors:** Josephine Borghi, Sharif Ismail, James Hollway, Rakhyun E. Kim, Joachim Sturmberg, Garrett Brown, Reinhard Mechler, Heinrich Volmink, Neil Spicer, Zaid Chalabi, Rachel Cassidy, Jeff Johnson, Anna Foss, Augustina Koduah, Christa Searle, Nadejda Komendantova, Agnes Semwanga, Suerie Moon

**Affiliations:** 1 Department of Global Health and Development, London School of Hygiene & Tropical Medicine, London, WC1H 9SH, UK; 2 Graduate Institute of International and Development Studies, Geneva, Switzerland; 3 Copernicus Institute of Sustainable Development, Utrecht University, Utrecht, The Netherlands; 4 School of Medicine and Public Health, University of Newcastle, Callaghan, Australia; 5 School of Politics and International Studies, University of Leeds, Leeds, UK; 6 International Institute for Applied Systems Analysis, Laxenberg, Austria; 7 Division of Health Systems and Public Health, Stellenbosch University, Stellenbosch, South Africa; 8 Institute for Environmental Design and Engineering, University College London., London, UK; 9 Faculty of Science, Technology, Engineering & Mathematics, The Open University, Milton Keynes, UK; 10 Department of Pharmacy Practice and Clinical Pharmacy, University of Ghana, Accra, Ghana; 11 Edinburgh Business School, Heriot Watt University, Edinburgh, UK; 12 Health Informatics Research Group, Makerere University, Kampala, Uganda

**Keywords:** complex adaptive system; global health system; health system

## Abstract

The global health system (GHS) is ill-equipped to deal with the increasing number of transnational challenges. The GHS needs reform to enhance global resilience to future risks to health. In this article we argue that the starting point for any reform must be conceptualizing and studying the GHS as a complex adaptive system (CAS) with a large and escalating number of interconnected global health actors that learn and adapt their behaviours in response to each other and changes in their environment. The GHS can be viewed as a multi-scalar, nested health system comprising all national health systems together with the global health architecture, in which behaviours are influenced by cross-scale interactions. However, current methods cannot adequately capture the dynamism or complexity of the GHS or quantify the effects of challenges or potential reform options. We provide an overview of a selection of systems thinking and complexity science methods available to researchers and highlight the numerous policy insights their application could yield.   We also discuss the challenges for researchers of applying these methods and for policy makers of digesting and acting upon them. We encourage application of a CAS approach to GHS research and policy making to help bolster resilience to future risks that transcend national boundaries and system scales.

## Introduction

Globalisation has increased interconnections across sectors and countries globally, intensifying transnational challenges that threaten global health, such as climate change, financial crises, anti-microbial resistance, and COVID-19. ^1^ However, the global health system (GHS), which has been defined as the transnational actors aiming to improve health and the governance, financing, and delivery systems within which they operate, ^2^ is ill-equipped to tackle these issues. In relation to COVID-19, for example, there remain profound inequities in the distribution of and access to vaccines, resulting from vaccine nationalism, inadequate global legal, pricing, procurement, allocation and delivery systems ^3^^,^^4^

There are renewed calls to re-imagine the GHS ^5^ to enhance global resilience to future transnational health risks. We argue that a key step towards this is improving the way we conceptualise and study the GHS. Health systems are recognised to be complex adaptive systems (CAS) and there is growing application of systems thinking and complexity science methods to help understand health system functionining ^6^^,^^7^ and to theorise, design and evaluate public health interventions to enhance health system performance at the national and sub-national levels. ^8^^,^^9^ The importance of a complexity lens to understanding and responding to the COVID-19 pandemic has also been highlighted, ^10^ together with the need for a paradigm shift away from a component-based view of the system towards a more dynamic view of how systems actually behave. ^11^ However, a CAS perspective has not typically been applied to the GHS within empirical research. In March 2021, a transdisciplinary group of public health, economics, health systems, global health governance, environmental science, systemic risk and resilience, mathematics, computer science, and engineering researchers with an interest in complex systems met to reflect on what we could learn from viewing the GHS as a CAS. This article draws on the workshop discussions and the participants’ research applying systems thinking and complexity science methods to national health systems and global systems outside of health (such as trade and environment) and policy engagement.

We begin by defining the features of a CAS, why the GHS can be considered a CAS and the limitations of current approaches to studying the GHS. We highlight how, by viewing it as a CAS, the GHS can be considered a multilevel system of systems, and show how four methods from systems thinking and complexity science could contribute to improving our understanding of the GHS and reforming it towards more effective global health outcomes.

## What is a complex adaptive system?

A system comprises of elements, interconnections (the way elements feed into and relate to each other), and a purpose. ^12^ A defining feature of a CAS is the focus on interconnections between system elements and in particular, the *adaptability* of those elements in response to changing conditions. ^11^ A CAS is self-organising and the properties of elements and the relationships between them can give rise to wholly new system behaviour, a property known as emergence. CASs are dynamic: constituent elements learn, adapt and evolve over time, shaped by interactions, past behaviours, and the external environment.

The GHS is a complex system ^13^ due to the large and escalating number of global health actors, ^2^ the varying and extensive networks linking them, ^14^ and the interconnections between health and other sectors, such as food, trade and the environment. ^15^ GHS behaviour demonstrates non-linear characteristics: small actions or inactions by specific actors can have large global level effects: vaccine hoarding by individual countries contributing to low vaccination availability in others. The GHS is also adaptive, responding to exogenous changes (the formation of the Access to COVID-19 Tools (ACT)-Accelerator Partnership in 2020 in response to COVID-19) or to changing characteristics of system elements (Germany increasing investment in the World Health Organization following United States threats of withdrawal). Furthermore, in hierarchical multi-level systems, both higher and lower levels, demonstrate layer dependent CAS properties. ^16^

## Limitations of current research and practice

Current research fails to consider the GHS as a CAS, which limits its potential to inform a reconfiguration of the GHS. Analysis of the GHS often focuses on specific system elements such as medicines, ^17^ or particular health actors ^18^; with less attention to the interactions and interconnections between elements/actors. Equally, there is fairly limited research on how the GHS influences national health systems, ^19^ the interconnections between national health systems, such as cross-border health systems ^20^ or regional health systems ^21^ despite their importance for pandemic threats, and the interconnections between health and other systems. ^22^

Epidemiological and economic studies grounded in game theory frameworks (the mathematical modeling of strategic interactions) have examined global solutions to transnational problems, such as cooperation on vaccine coverage for disease eradication. ^23^^–^^25^ Evolutionary game theory was recently used to analyze the factors influencing active cooperative governance and proposed a series of recommendations for promoting international cooperation in relation to COVID-19. ^26^ However, such models often assume actors are rational utility maximisers and the global system is in equilibrium, overlooking the evolving and dynamic nature of the GHS. Political economy approaches can provide insight into power dynamics, values, economic and political interests shaping global health institutions and policy ^27^ together with historical factors underpinning these, but have difficulty quantifying their effects or modelling the consequences of potential reform options.

The effects of framing GHS behaviour without reference to complex adaptive behaviour can be seen in COVID-19 policy responses which have tended to overlook ways in which interventions would interact with existing health system structures. For example, calls for a Global Health Threats Fund ^28^ have not considered the administrative costs (and relative efficiency) of creating and running a new global fund, nor how the fund would interact with an already highly fragmented global financing landscape and the consequences for domestic financing arrangements. Equally, efforts to estimate the costs of pandemic preparedness at the global, regional and national levels and to identify mechanisms to finance these, often ignore the interconnections between global, regional and national systems, and how investments at one level will affect costs and resource needs at other levels. ^29^

## The value of a complex adaptive systems approach

**Figure 1.**
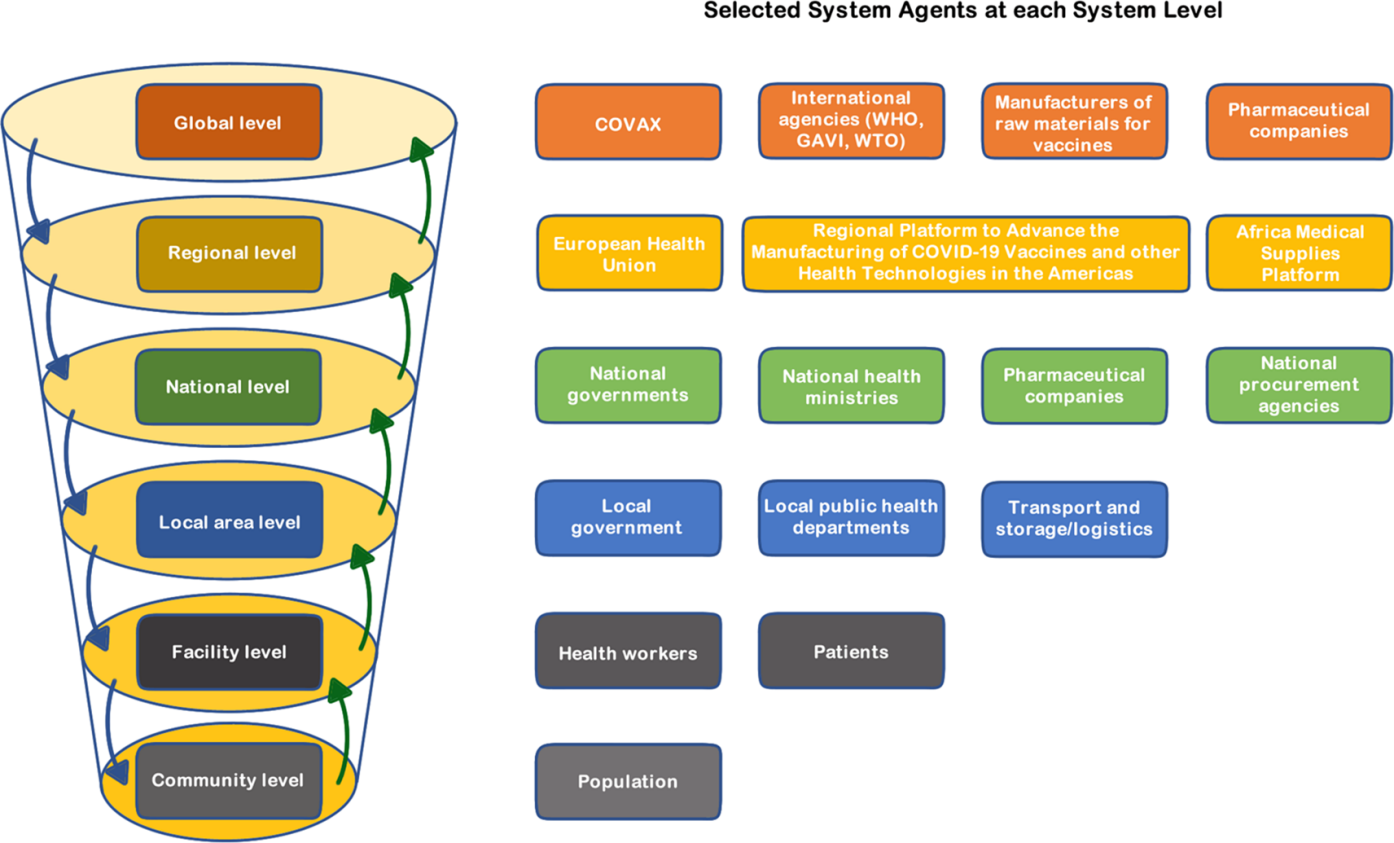
An illustrative simplified multi-level systems view of the COVID-19 vaccination subsystem. Note: System behaviour responds to bottom up and top-down influences, with higher level entities (global actors (pharmaceutical companies) and initiatives (COVAX)) influencing COVID-19 vaccine production, affordability and deployment options of lower-level agents; and actions of lower level agents (e.g. patients uptake or not of vaccines) influencing global responses. ^35^

A CAS approach can be used to study the supra-national actors and systems that support global health consistent with the definition of the GHS provided earlier. ^2^ Through a CAS lens, the GHS can also be conceptualised as a multi-level health system, a continuum of health systems from local to global, comprising all the national systems globally together with the supranational global ‘health system’ architecture, as illustrated in the simplified configuration in Figure 1. Taking a multilevel systems view of the GHS is helpful when a system goal is influenced by actions across system levels, as is the case for COVID-19 vaccination equity. For example, resolving vaccine production and allocation constraints requires global level actions ^30^ (e.g. patent and pricing regulations, contract manufacturing arrangements), while affordability and deployment constraints require actions at the national (e.g. funding/budgetary allocations, transport, storage, logistics) and local levels (overcoming vaccine hesitancy to enable population uptake ^31^).

A CAS lens further recognizes the interconnections between national health systems which may be relevant to understanding how, for example, allocation of COVID-19 vaccines is affected by inter-country transfers of vaccines, ^32^ or how in-country deployment is shaped by policies to enhance supply chains and vaccination uptake in neighboring countries.

The importance of taking a multi-scale approach to health is only slowly starting to be recognised in relation to COVID-19, ^29^ while multi-level governance systems and the study of cross-scale interactions have long been the subject of climate research. ^30^

Beyond an alternate conceptualization of the GHS, as we set out below, a CAS approach can help to visualize and understand system structure and dynamics, quantify the effect of a problem, and predict system behaviour in response to exogenous shocks or policy reforms. Importantly such an approach considers how system structure and the interaction and behaviour of elements within the system can result in the emergence of internal vulnerabilities rather than assuming that system failures are solely due to events outside the system. ^11^ This helps move towards an *ex-ante* approach of understanding and reducing risk. ^33^ By conceptualizing interconnections and offering tools enabling their study, a CAS lens could potentially offer new insights on global and local system constraints, emergent systemic risks, and feasible and effective reform strategies for policy and practice.

## Operationalising a complex adaptive systems approach for the global health system

In this section we illustrate the potential of a series of systems thinking and complexity science approaches to improve understanding of the GHS by offering new and complementary insights relative to existing methods. Rather than attempting to model the GHS in its entirety, these sense-making tools instead focus on establishing what contributes to a particular system behaviour (or outcome) of interest, and how.

### Determining system boundaries

A critical first step to operationalizing a CAS approach is determining the boundaries of the system of interest – i.e. to consider what is relevant to the behaviour of interest and its spatial and temporal scope, while acknowledging what is external to the system but may nevertheless influence system functioning. System boundaries depend on the goal of the system ^34^ (or the problem within the system one is trying to address) and are ultimately arbitrary. If drawn too narrowly, important feedback loops may be missed; if drawn too broadly the analysis becomes meaningless as to the problem addressed. ^35^ Thus boundaries can be determined through stakeholder discussion using methods such as group model building, ^36^ which develop a shared understanding of the system, or use of a conceptual frame. ^37^ The “critical systems” tradition in systems thinking, and more specifically critical systems heuristics, provides a framework to problematise researcher and stakeholder assumptions about system boundaries (and system goals), and encourage critical reflection on what these imply. ^38^ The goal and the level of the system to which it applies will determine who to engage in boundary setting (typically those influencing the goal and those affected by it). Existing frameworks can facilitate researcher and policy reflection about goals and system boundaries for the GHS. ^1^^,^^2^^,^^39^

We now focus on four approaches that have been applied to the study of health systems at the national and sub-national levels and have been used to study global systems in other sectors: causal loop diagrams, system dynamic modelling, agent based modelling and network analysis. We also introduce the concept of global system science as an approach to studying the interaction between global systems.

### Causal loop diagrams

**Figure 2.**
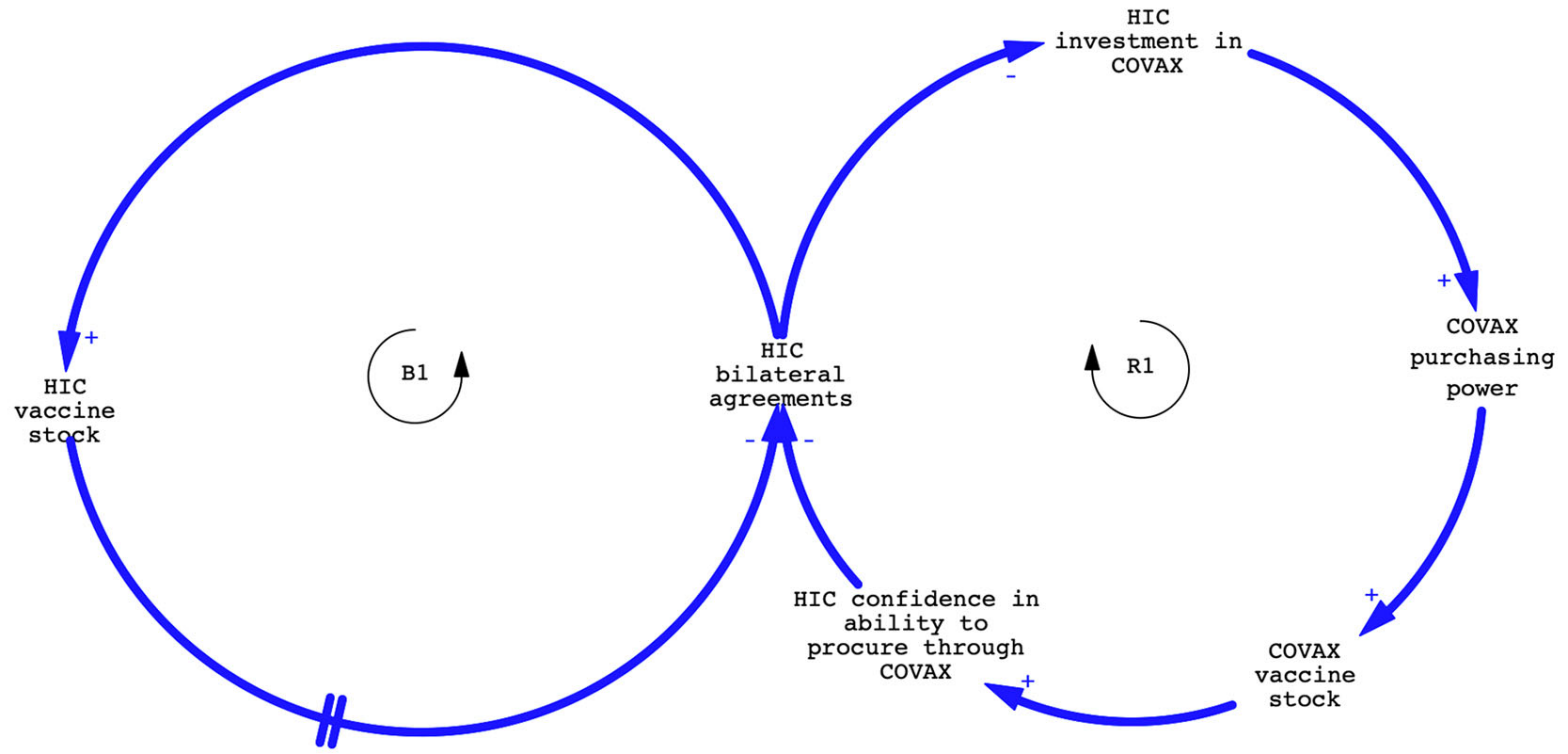
Simplified illustration of a reinforcing and balancing loop in relation to HICs entering into bilateral deals to secure COVID-19 vaccines. Note to Figure:
1.Principles of balancing and reinforcing feedback loops: “+” indicates a change in the same direction, “-”in the opposite direction to the change in the variable at the start of an arrow. A reinforcing (R) loop has an even number, a balancing (B) loop an uneven number of “-”relationships. “//” indicates a delay.2.The principle context of COVAX has been omitted for simplicity reasons, and only the impact of making bilateral agreements has been illustrated.

Mapping techniques derived from systems thinking such as causal loop diagrams (CLDs), can be used by researchers or policy makers to identify and visually represent causal relationships between elements within the GHS and determine how these influence system behaviour and thereby its ability to achieve desired outcomes. CLDs illustrate feedback loops, such as a reinforcing loop (R), where a relationship shows an amplified effect over time, or a balancing loop (B), reflecting stabilizing behaviour. By identifying feedback loops reinforcing problem behaviours, and critical elements that underpin system functioning, ^40^ CLDs can be used to determine leverage points, where interventions should be focused to enhance system performance and thereby inform policy development and prioritization. Figure 2 provides a simplified illustratation of a causal loop representation showing how early on, high income country (HIC) pursuit of bilateral deals undermined COVAX as HICs were unwilling to procure vaccines through COVAX. This reduced the funds available to COVAX and its purchasing power, limiting the ability of COVAX to procure COVID-19 vaccines, reinforcing the pursuit of bilateral deals by HICs (R1). The impetus to bilateral deals by HICs was in theory constrained by rising HIC vaccine stock (B1) but in the early phases of the global vaccine rollout, this feedback loop was in reality weak. It did eventually strengthen, helping to trigger an increase in HIC investment in COVAX.

There is growing use of CLDs to study national health systems and their response to policy reforms ^40^^,^^41^ and exogenous shocks ^42^ to identify factors shaping vaccine demand, supply and trust. ^43^^,^^44^ CLDs have great potential to address GHS issues by apriori examining the implication of planned policy shifts.

### System dynamic modelling

Adding data and functional relationships to the structure generated through CLDs creates system dynamics models (SDM) which use stocks and flows to simulate *aggregate* system behaviours over time and quantify the relationship and dynamic interactions between system elements and how changes in the stock of one element, such as financing, would affect other elements, such as the stock of vaccines. In this way, SDM can quantify the effect of an exogenous shock, such as COVID-19, or the impact of a mitigating strategy or policy intervenention on an outcome of interest. ^45^

### Network analysis

A network consists of a set of nodes and links connecting them. Network analysis techniques can map, measure, and model the network structure or topology of the GHS and its evolution over time. They can be used to determine whether the structure is polycentric or fragmented, as has been done in relation to global environmental governance, ^46^ or understand how that structure came to be formed in the first place. Network analysis can also be used to determine systemic risk and explore how reshaping the system’s network topology might enhance its resilience. Quantifying the interdependencies between system nodes, and the relative contribution of these to overall systemic risk allows identification of nodes whose failure due to their size or interconnectedness could trigger ripple and cascading system effects. ^33^^,^^47^

**Figure 3.**
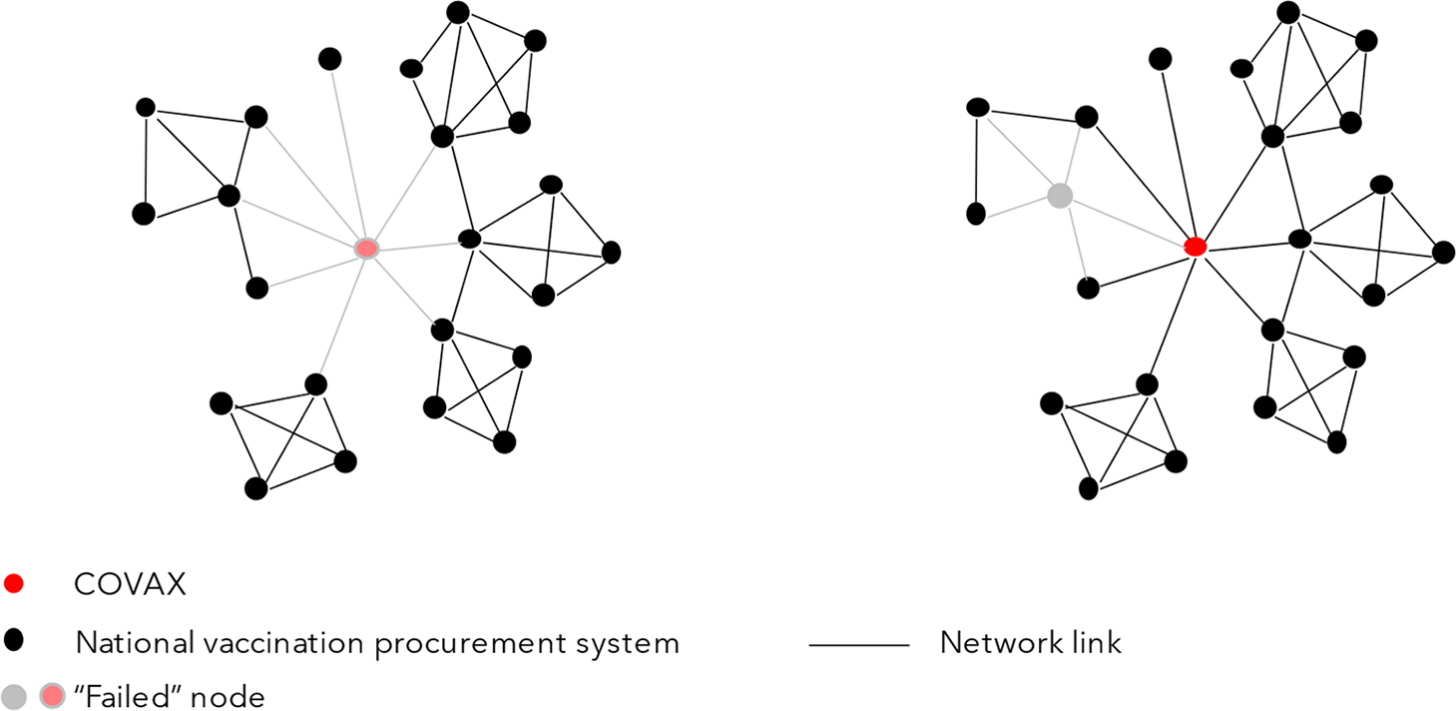
Illustration of a network structure under two scenarios, showing how the failure of a single node may lead to very different systemic outcomes depending on its position in the network. The network on the right remains intact whereas the network on the left disintegrates into four fragments.

Figure 3 provides a simple network schematic highlighting how the failure of a single node may lead to very different systemic outcomes depending on its position in the network. Let us assume that the central node in red is COVAX and the other nodes are national COVID-19 vaccination procurement systems. The left panel illustrates the situation where COVAX fails, as it did early on in vaccine deployment when the limited stock of vaccines held by COVAX contributed to limited allocation of vaccines to LMICs. The right panel illustrates the more limited consequences when it is a more peripheral node that fails, such as when allocation problems chiefly arose at the country-level, due to weak health systems and demand constraints, but without necessarily affecting other countries’ access. This highlights some of the advantages and disadvantages of a reasonably centralized topology.

Network analysis is increasingly used to study national and local health systems, ^48^ including the performance of facility networks and provider networks within health facilities. Its application at the global level has been less frequent, but includes the study of the global spread of COVID-19 vaccine misinformation ^49^ or vaccine patents which pinpointed constraints to technology transfer. ^50^ There is thus further potential to apply these methods to other aspects of the GHS and governance as has been done within the European Union. ^51^

### Agent-based modelling

With agent-based models (ABM), the system is modelled as a collection of automous and heterogenous agents that interact and make decisions based on their attributes and a set of rules governing their behaviour. ^52^ ABM provides an exploration of the dynamic behaviour of the system resulting from the interactions between agents within the GHS (such as non-governmental organizations, corporations, governments, multilaterals, health facilities, or individuals) and with their external environment. Agents can learn and evolve, allowing for unanticipated effects to emerge from the agents’ characteristics and behaviours within a system. There is growing application of ABM to study national health systems, for example, identifying design features within a provider payment mechanism to achieve maximum financial and quality outcomes. ^53^ ABM has been used to study global systems outside of health, examining constraints to global governance and opportunities for greater coordination between nation states ^54^ and could be potentially used to support more effective global health governance. For example, ABM could be used to identify which incentive structure would be most effective in getting high income countries’ vaccine production, allocation, and deployment to align with the interests of the global South.

While these methods can be used in isolation, they can also be used jointly, with potential to build hybrid SDM and ABM models, ^7^ using SDM as an input to ABM and vice versa; and linking network analysis and ABM. ^55^^,^^56^

### Global system science

Moving beyond the GHS to study interactions and interdependencies with other global systems, global system science examines system behaviour at the global scale, involving complexity science, informatics, policy implementation and public engagement ^57^ and could be extended to global health, for example, examining the effect of trade, environment and communication systems on the GHS. ^1^

## Challenges

Despite the advantages, there are a number of challenges in applying a CAS lens to the GHS. When mapping or modelling any system, it can be a struggle to get the right balance between capturing enough detail to convey the complexity of the system without obfuscating major patterns. Given the scale of the global system and potential for cross-scale influences, containing complexity to enable meaningful insights is not without challenge. However, CAS studies of other global systems provide reassurance this can be achieved for the GHS. ^14^^,^^46^

In addition, models typically rely on data that may be inaccessible, incomplete or inconsistent across geographies, requiring assumptions and reliance on researcher values with the inherent risk of bias. Engagement with a diverse set of stakeholders is critical in defining the problem/s to be addressed and making boundary judgements, building a qualitative understanding of the system through a visual map, before iteratively building, parameterising, and validating quantitative models, and thereby increasing their acceptability and validity. However, stakeholders may differ in their understanding of the system and in its perceived goals, which can create challenges in building a unified model of the system.

Furthermore, there are limits to the methods reviewed. For example, SDM have not, to date, been used extensively to consider power dynamics, nor the economic consequences of reforms, and would need to be complemented and informed by political economy and policy analysis to assess the political acceptability of potential reform options, and economic models, to determine their financial and macro-economic implications. Studying the GHS as a CAS will require an interdisciplinary research effort to integrate knowledge on global health governance and policy, health systems, social science, mathematics and engineering, requiring collaboration across research departments and institutions, and the development of a shared lexicon and understanding of related concepts and methods.

From a policy perspective, increased awareness and understanding of systems and complexity thinking ^58^ will be needed to encourage the use of complexity science models among policy makers and the uptake of resulting research. ^59^ Evidence of growing policy engagement with systems thinking approaches as evidenced by guidance documents produced by government and intergovernmental organisations is encouraging. ^60^^,^^61^ Furthermore, the political interests of actors and power imbalances within the GHS could limit the global community's willingness to implement reform recommendations resulting from CAS research on the GHS. However, COVID-19 has created recognition of the need for reform and resulted in initiatives to enhance global health solidarity, which provides an avenue to which systems thinking and complexity science could contribute. ^5^^,^^62^^,^^63^

## Conclusions

We have shown that the GHS embodies the properties of a nested multi-scale CAS and that greater researcher and policy engagement with CAS tools could offer new insights into system constraints and potential leverage points to better achieve GHS goals. We encourage application of system dynamics, agent based modelling, network analysis and other systems and complexity science methods to inform the effective design of global systems that promote global public goods for health to tackle future transnational challenges such as pandemics and climate change. Arising proposals will need to be complemented by political economy studies of the feasibility and potential acceptability of reform options, and economic studies of their financial implications. Recognizing the complexity of the GHS and being able to analyze and predict the performance of the GHS is an essential precondition for reforming and strengthening it in an impactful way.

## Data availability

No data are associated with this article.
