# Supplementary material for: Viewing the global health system as a complex adaptive system – implications for research and practice
Source: F1000Res. 2022 Oct 7;11:1147. [Version 1] doi: 10.12688/f1000research.126201.1 (PMC10432894; doi:10.12688/f1000research.126201.1)
Supplement: Peer review history

# Peer review: Reviewer response for version 1

**DOI:** 10.5256/f1000research.138585.r173947

**Published:** 2023-08-16

**Authors:** Corinna Elsenbroich

**Affiliations:** 1 MRC/CSO Social and Public Health Sciences Unit, School of Health and Wellbeing, University of Glasgow, Glasgow, Scotland, UK

This is a really good statement piece about systems methods and how they can help to improve the Global Health System. The authors describe the methods in sufficient detail and with enough motivation to let the reader understand the importance of a new approach. My one suggestion is to maybe extend the problem of data and evidence a bit more in the Challenges section. Whilst the authors talk about that data can be inconsistent across countries or not collected to date, there are even worse problems with some data not being available in principle (some things that are modelled in complex systems models might not be quantifiable). On evidence, in particular in health there is a strong focus on "gold standard evidence" and CSM will not provide this kind of evidence.

Is the rationale for developing the new method (or application) clearly explained?

Yes

Is the description of the method technically sound?

Yes

Are the conclusions about the method and its performance adequately supported by the findings presented in the article?

Yes

If any results are presented, are all the source data underlying the results available to ensure full reproducibility?

No source data required

Are sufficient details provided to allow replication of the method development and its use by others?

No

Reviewer Expertise:

Complexity Social Science

I confirm that I have read this submission and believe that I have an appropriate level of expertise to confirm that it is of an acceptable scientific standard.

# Peer review: Reviewer response for version 1

**DOI:** 10.5256/f1000research.138585.r166534

**Published:** 2023-04-18

**Authors:** Alastair Ager

**Affiliations:** 1 Institute for Global Health and Development, Queen Margaret University, Edinburgh, Scotland, UK; 2 Mailman School of Public Health, Columbia University, New York, New York, USA

This paper articulates the argument that institutions and actors active in the field of global health be conceived as part of a global health system that operates as a form of ‘complex adaptive system’ (CAS). The authors go on to outline a number of analytic approaches associated with the ‘systems thinking’ of value in understanding the behavior of CAS - including causal loop modelling, system dynamic analysis, network analysis and agent-based modelling - and to consider both the value of and challenges associated with each in their application if the field of global health.

 The authors particularly draw upon the experience of the COVID-19 pandemic – and responses such as the COVAX vaccine initiative – to illustrate the value of seeing the behavior of actors and institutions within the global health system as interdependent. The paper usefully draws attention to complexity being associated with both the multi-scalar nature of analysis required in any context (e.g. from communities through to national Ministries) and the reality of globalization that sees national and international actors connected though a wide variety of multilateral, bilateral and commercial relationships. By illustration, the authors clearly illustrate interdependence and systems effects, although the claim that a complex adaptive system approach can “predict system behavior in response to exogenous shocks or policy reforms” is perhaps best understood as an aspiration at this stage.

 The brief introductions to systems methods are effective in illustrating the character and potential of each, again with appropriate illustration provided. The paper thus serves as a useful ‘reader’ compiling examples of recent applications of these methods in addressing health systems challenges. While the simplified examples are warranted – supporting a clear, concise narrative for the paper – they fall some way short of fulfilling the vision established at the start of the paper for anticipating adaptations such as emergence and non-linear characteristics in a manner that would inform health sector policy and practice.

 The challenges with which the paper closes – the balance of detail and comprehensibility in analyses, data availability, measurement of influential but ‘hard-to-operationalize variables such as power and trust, policymaker openness to complexity – are, in my view, indeed the key challenges in advancing such forms of analysis at this time. However, they are addressable, and the paper serves as a useful call for greater engagement with this burgeoning field of work in informing – in increasingly concrete and evidence-informed ways – the formulation of global health policy and strategy.

Is the rationale for developing the new method (or application) clearly explained?

Yes

Is the description of the method technically sound?

Yes

Are the conclusions about the method and its performance adequately supported by the findings presented in the article?

Partly

If any results are presented, are all the source data underlying the results available to ensure full reproducibility?

No source data required

Are sufficient details provided to allow replication of the method development and its use by others?

Partly

Reviewer Expertise:

Health systems resilience; Global health policy; Evaluation of humanitarian action.

I confirm that I have read this submission and believe that I have an appropriate level of expertise to confirm that it is of an acceptable scientific standard.
